# Plasma metabolomics in tuberculosis patients with and without concurrent type 2 diabetes at diagnosis and during antibiotic treatment

**DOI:** 10.1038/s41598-019-54983-5

**Published:** 2019-12-10

**Authors:** Frank Vrieling, Bachti Alisjahbana, Edhyana Sahiratmadja, Reinout van Crevel, Amy C. Harms, Thomas Hankemeier, Tom H. M. Ottenhoff, Simone A. Joosten

**Affiliations:** 1 Department of Infectious Diseases, Leiden University Medical Centre, Leiden, The Netherlands; 2 Department of Internal Medicine, Hasan Sadikin General Hospital, Bandung, Indonesia; 3 Infectious Disease Research Center, Faculty of Medicine, Universitas Padjadjaran, Bandung, Indonesia; 4 Department of Internal Medicine and Radboud Center for Infectious Diseases, Radboud Institute for Health Sciences (RIHS), Radboud University Medical Center, Nijmegen, The Netherlands; 5 Systems Biomedicine and Pharmacology, Leiden Academic Centre for Drug Research, Leiden University, Leiden, The Netherlands

**Keywords:** Tuberculosis, Diabetes

## Abstract

Tuberculosis (TB) and type 2 diabetes mellitus (DM), a major TB risk factor, are both accompanied by marked alterations in metabolic processes. Dissecting the specific metabolic changes induced by disease through metabolomics has shown potential to improve our understanding of relevant pathophysiological mechanisms of disease, which could lead to improved treatment. Targeted tandem liquid chromatography–mass spectrometry (LC-MS/MS) was used to compare amine and acylcarnitine levels in plasma samples of patients with TB or TB-DM from Indonesia at time of diagnosis and during antibiotic treatment. Partial least squares discrimination analysis (PLS-DA) showed good separation of patient groups. Amine levels were strongly altered in both disease groups compared to healthy controls, including low concentrations of citrulline and ornithine. Several amino acid ratios discriminated TB from controls (phenylalanine/histidine; citrulline/arginine; kynurenine/tryptophan), possibly reflecting changes in indoleamine-pyrrole 2,3-dioxygenase (IDO) and nitric oxide synthase (NOS) activity. Choline, glycine, serine, threonine and homoserine levels were lower in TB-DM compared to TB, and, in contrast to other analytes, did not normalize to healthy control levels during antibiotic treatment. Our results not only provide important validation of previous studies but also identify novel biomarkers, and significantly enhance our understanding of metabolic changes in human TB and TB-DM.

## Introduction

Tuberculosis (TB) is a severe infectious disease which mostly affects the lungs and is caused by *Mycobacterium tuberculosis*. In 2016, 10.4 million people were newly diagnosed with TB and 1.7 million individuals died as a result of TB, ranking TB as the 10^th^ leading cause of death worldwide^1^. Over recent years, type 2 diabetes mellitus (DM) has been recognized as an important risk factor for TB development and reduced success of TB treatment^2–4^. It is currently estimated that 15% of global TB cases can be attributed to concurrent TB-DM^5^. The number of people living with DM worldwide is estimated to increase by 48% in 2045, especially in low- and middle-income countries, where TB is endemic, due to changes in lifestyle associated with economic development and urbanization^6^. Therefore a better understanding of the characteristics governing TB in the context of DM comorbidity is crucial for deciphering their combined pathophysiology and ultimately improved treatment.

Both TB and DM are accompanied by marked metabolic changes: TB progression is associated with the development of wasting syndrome, a nutritional state during which the combination of increased energy expenditure necessary to combat the infection and decreased food-intake leads to severe weight loss and wasting of muscle tissue, whereas hyperglycemia and hyperlipidemia are major hallmarks of DM. We recently showed that TB-DM patients display metabolic characteristics of both diseases as determined by ^1^H-Nuclear Magnetic Resonance (NMR) plasma lipid profiling^7^. Metabolomics, defined as the comprehensive analysis of small molecule intermediates of metabolism within a biological system which together form the metabolome, has developed into a powerful approach to study potential perturbations of metabolic homeostasis caused by disease. The use of metabolomics has resulted in the successful identification of small molecule metabolite biomarkers for various illnesses, including Alzheimer’s disease^8^, various forms of cancer^9^, and diabetes^10^. A number of studies have used metabolomics to identify biomarkers for TB in both serum and urine^11^. More recently, a prognostic metabolic biosignature with good predictive power for TB progression was developed^12^. However, further validation of many of these biomarker candidates has not been performed in independent studies or in the presence of clinically relevant comorbidities such as DM.

Here, we performed targeted metabolomics to investigate amine and acylcarnitine levels in plasma samples of TB patients with or without DM and healthy endemic controls. Acylcarnitines are intermediates of fatty acid and amino acid oxidation which may be involved in early insulin resistance^10^. Furthermore, the metabolic profiles of both TB and TB-DM patients were followed longitudinally during TB treatment to analyze possible effects of antibiotic TB treatment on metabolite biomarkers. We find that TB and TB-DM have both shared and unique effects on patient plasma metabolic profiles, including marked changes in metabolites involved in the urea cycle, indoleamine 2,3-dioxygenase (IDO) signaling and liver function, of which the majority normalized to healthy control levels during the course of antibiotic treatment. The results of this study not only confirm and validate key findings from previous metabolomics studies on TB in a geographically and genetically distinct population, but also propose novel biomarker candidates for TB and TB-DM.

## Results

### Study population

In total, metabolite concentrations were measured in plasma samples from 48 TB patients, 20 TB-DM patients and 48 healthy controls (HC). HC had a similar age but higher body weight compared to TB patients without DM; diabetic TB patients were older and had a higher BMI compared with non-diabetic TB patients (Table 1). No significant differences were present between the groups based on sex, ethnicity, current smoking status or severity of TB scored on chest x-rays (CXR).

**Table 1:** Patients’ clinical characteristics according to disease group (n = 116).

	HC n = 48	TB n = 49	TB-DM n = 19	*p*-value
Sex (male/female)	24/24	23/26	13/6	0.269
Age (years)	29.0 ± 9.1	29.4 ± 9.1	48.3 ± 8.5	<0.001
BMI (kg/m^2^)	22.9 ± 4.0	17.3 ± 2.1	20.5 ± 2.9	<0.001
Fasting blood glucose (mg/dl)	80.2 ± 9.1	80.7 ± 13.6	218.5 ± 76.8	<0.001
Smoking (currently)	20/48 (41.7%)	19/49 (38.8%)	9/19 (47.4%)	0.811
CXR score (mild/advanced)	na	21/28	10/9	0.468
Ethnicity:				0.650
Betawi	10/48 (20.8%)	11/49 (22.4%)	3/19 (15.8%)	
Jawa	17/48 (35.4%)	13/49 (26.5%)	6/19 (31.6%)	
Sunda	8/48 (16.7%)	12/49 (24.5%)	4/19 (21.1%)	
Mixed	10/48 (20.8%)	11/49 (22.4%)	3/19 (15.8%)	
Other	3/48 (6.3%)	2/49 (4.1%)	3/19 (15.8%)	

Data is presented as percentage of total (%) or mean ± SD. BMI = Body Mass Index, CXR = Chest X-ray Radiograph.

**Figure 1:**
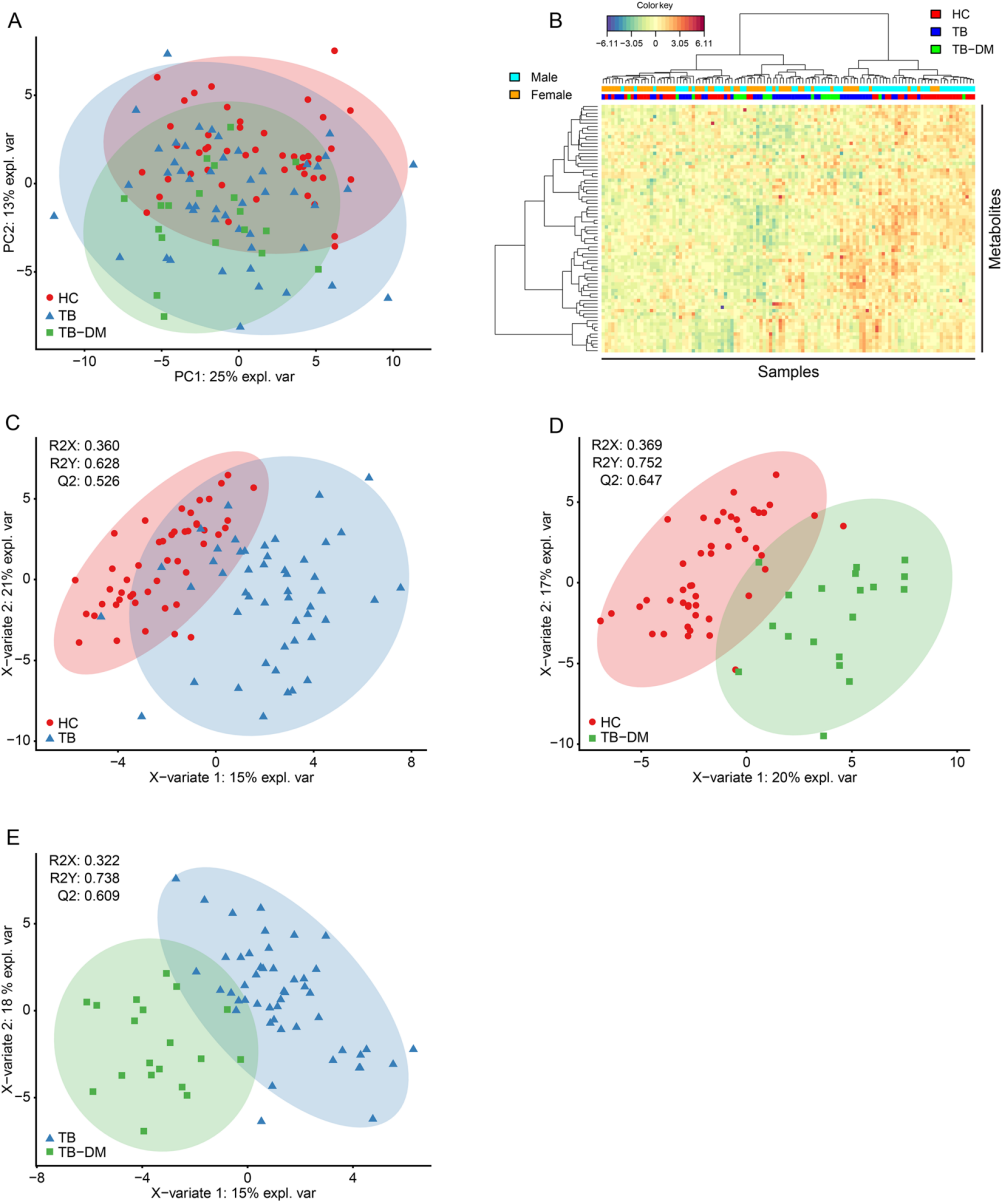
TB/TB-DM status result in distinct metabolic profiles. (**A**) Score plot of the first two principal components of a PCA model built on the entire dataset and color coded by group with confidence ellipses. HC are displayed as red dots, TB patients as blue triangles and TB-DM patients as green squares. (**B**) Two-way hierarchical clustering analysis by Euclidean distance with Ward’s method. Samples are annotated by disease group and sex: HC (red), TB patients (blue) and TB-DM patients (green), male (cyan), female (orange). (**C**–**E**) Score plots of PLS-DA models for TB vs HC (**C**), TB-DM vs HC (**D**) and TB-DM vs TB (**E**). HC are displayed as red dots, TB patients as blue triangles and TB-DM patients as green squares. PLS-DA evaluation criteria (R2X, R2Y, Q2) are displayed for each model.

First, a principal component analysis (PCA) model was built to visualize differences between disease groups based on the entire dataset, which consisted of four components explaining 54% of total variance. The score plot of the first two components (explaining 25% and 13% of total variance, respectively) is displayed in Fig. 1A. While disease status (HC, TB or TB-DM) accounted for a proportion of the total variance, no complete separation was observed between the three groups. However, sex differences also comprised a considerable source of data variance (Fig. S1A). These results were corroborated by hierarchical clustering analysis which showed incomplete clustering based on either sex or disease group status (Fig. 1B). To correct for the effect of sex, a multilevel PCA model was built^13^ to separate the “within-sex” from the “between-sex” data variation. The multilevel model improved the discriminatory capacity based on disease group (Fig. S1B), while neither sex nor smoking status contributed to data variance. Finally, partial-least squares discrimination analysis (PLS-DA) models were fitted for each disease group comparison and the resulting score plots and cross-validated quality metrics for model predictive ability (Q2) and explained variance (R2X & R2Y) are displayed in Fig. 1C–E. All models showed high goodness of fit and predictive ability as indicated by R2Y and Q2 scores of >0.5, and the resulting score plots showed relatively good clustering and separation of samples based on disease group. Taken together, TB and TB-DM status were found to be major contributors to data variance based on the entire metabolomic dataset, and adjusting for differences in sex was of importance for further analysis.

### Patients with TB and TB-DM have distinct metabolic profiles

**Figure 2:**
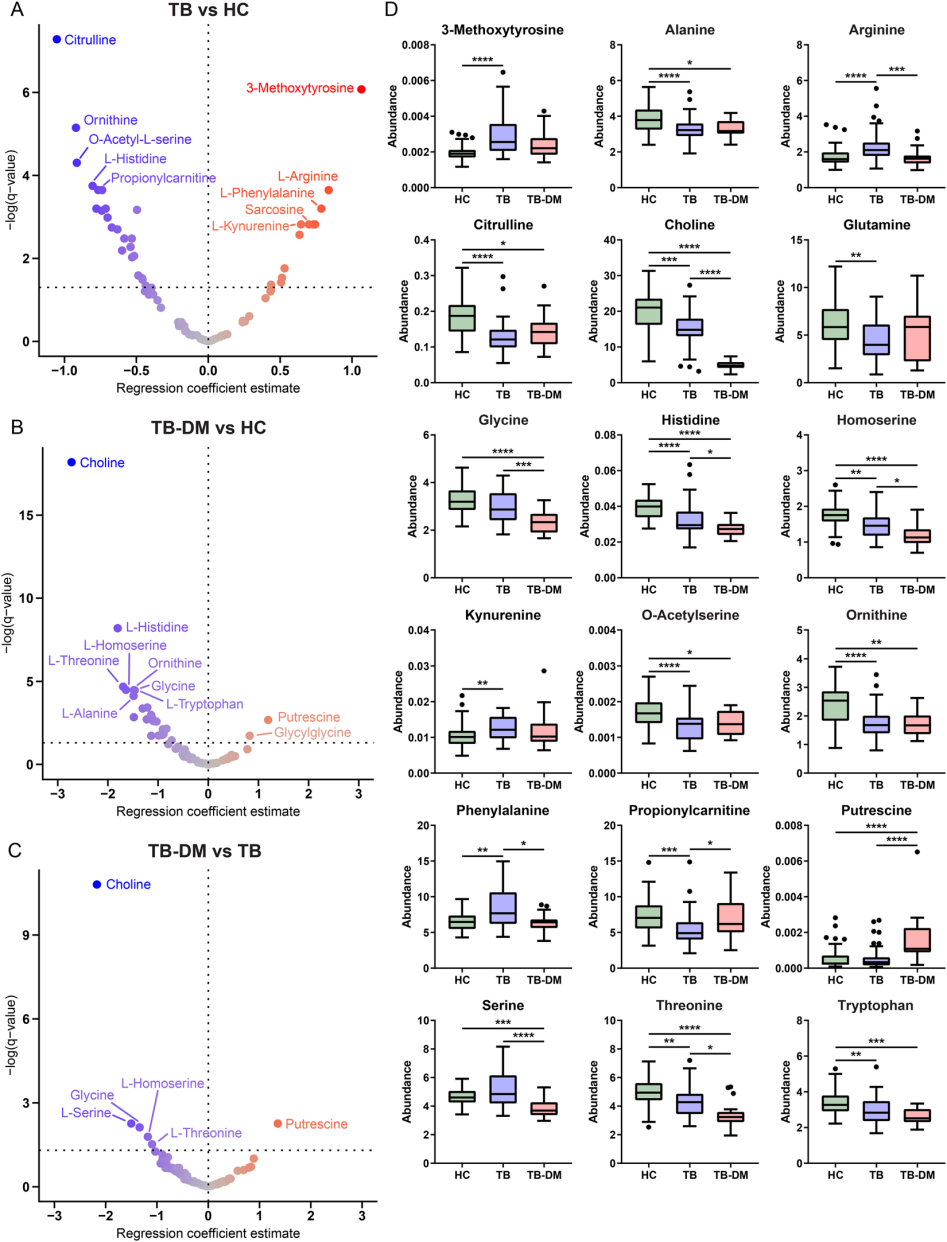
TB and TB-DM greatly impact patient plasma metabolite levels. Multiple linear regression models were fitted for each between disease group comparison, and resulting -log-transformed *p*-values (*q*-values) are plotted against the regression coefficient estimate for each metabolite: TB vs HC (**A**), TB-DM vs HC (**B**), TB-DM vs TB. (**C**) Each dot represents an individual metabolite. Dot color represents direction and size of the regression coefficient. The significance threshold (*q* = 0.05) is displayed as a horizontal dotted line. (**D**) Absolute abundance of individual metabolites per group displayed as Tukey’s boxplots. Significance differences between HC (n = 48), TB (n = 48) and TB-DM (n = 20) groups were determined by Kruskal-Wallis test with post-hoc Dunn’s test. *p = 0.05, **p = 0.01, ***p = 0.001, ****p = 0.0001.

In total, levels of 31/53 amines (58.5%) and 5/21 acylcarnitines (23.8%) were significantly different in TB patients compared to HC based on a linear regression model (Fig. 2A). Medians with interquartile ranges of all measurements and their resulting *q*-values can be found in Supplementary Table 1. Volcano plots of regression model statistics versus metabolite log2-transformed fold changes are depicted in Fig. S2. TB was strongly associated with low levels of citrulline and ornithine, both central amino acids of the urea cycle (Fig. 2D), whereas levels of arginine and aspartic acid, two other important intermediates in the urea cycle, were higher in TB patients. Furthermore, levels of histidine were significantly reduced, while those for phenylalanine were increased in TB patients, a finding which is congruent with previous metabolomics analyses^7^. The metabolite with the strongest positive association with TB was 3-methoxytyrosine, a metabolite of levodopa which is mostly associated with aromatic L-amino acid decarboxylase (AADC) deficiency. Other notable changes included significantly lower levels of tryptophan and higher plasma concentrations of kynurenine, two metabolites which are part of the immunoregulatory enzyme IDO pathway.

The metabolite profile of TB-DM patients (Fig. 2B) was mostly characterized by low levels of amines when compared to HC, while only two metabolites, putrescine and glycylglycine, were significantly elevated. Similar to TB patients, TB-DM was associated with low levels of citrulline, histidine, ornithine and tryptophan, among others. However, the most notable difference was the exceptionally low average concentration of choline compared to HC (*q* = 6.45E^−19^), an effect which was magnitudes stronger than observed in TB patients without DM (Fig. 2C). Similarly, levels of serine, homoserine, glycine and threonine, were significantly lower in TB-DM patient plasma compared to TB patients, as well as to HC. These results are congruent with earlier studies describing decreased glycine, serine and threonine levels during DM^14,15^.

### Metabolite ratios show potential for TB and TB-DM classification

**Figure 3:**
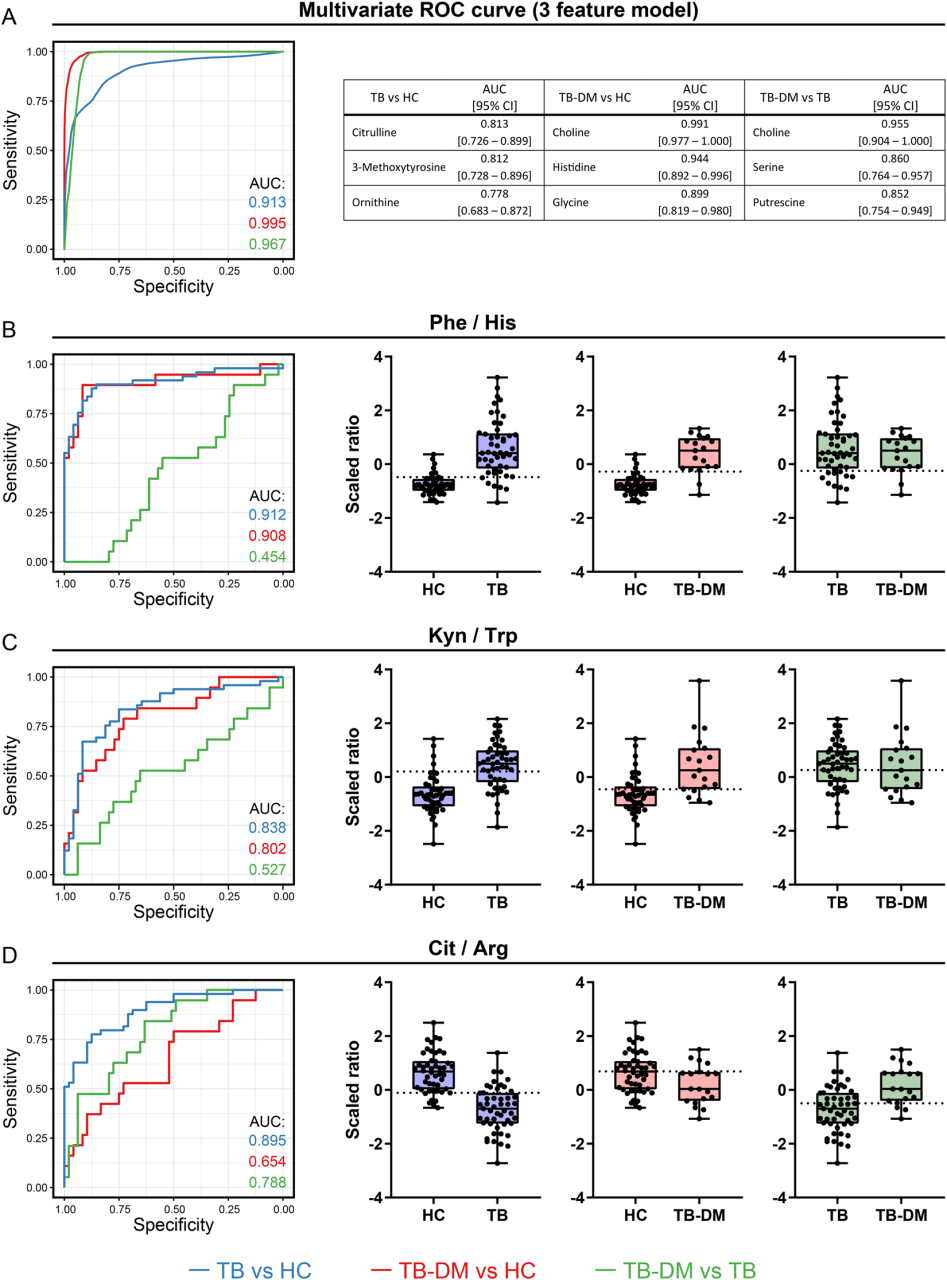
Biomarker analysis. ROC curves and AUCs were determined for each metabolite and disease group comparison: TB vs HC (blue), TB-DM vs HC (red) and TB-DM vs TB (green). (**A**) The three metabolites with the highest AUCs per comparison were combined in a 3-feature model and corresponding multivariate ROC curves were fitted by linear support vector machine algorithm. (**B**–**D**) ROC curves and boxplots of the following metabolite ratios are shown: phenylalanine/histidine (Phe/His) (**B**), kynurenine/tryptophan (Kyn/Trp) (**C**) and citrulline/arginine (Cit/Arg) (**D**). Each dot represents an individual patient and the optimal cut-off as determined by Youden’s statistic is displayed as a horizontal dotted line.

In order to evaluate their potential as metabolic biomarkers for TB or TB-DM, receiver operating characteristic (ROC) curves were plotted per metabolite for each disease group comparison and the resulting AUC values were calculated (Supplementary Table 1). The three metabolites with the highest AUC values were subsequently incorporated into multivariate signatures and their classification effectiveness was tested by a linear support vector machines (SVM) machine learning algorithm (Fig. 3A). Citrulline, 3-methoxytyrosine and arginine were the individual metabolites with the best classification capacity for TB versus HC, which was further improved by their inclusion in a multivariate signature (AUC: 0.913 [0.818–0.978]). As expected, choline was the superior biomarker for TB-DM versus HC from our dataset (AUC: 0.991 [0.977–1.000]), followed by histidine and glycine. Choline, serine and putrescine showed the highest potential for discriminating TB-DM from TB patients. Incorporation into cross-validated multivariate models in these cases resulted in similar AUC values (TB-DM vs HC: 0.995 [0.981–1.000]; TB-DM vs TB: 0.967 [0.940–0.998]). While some of our data validate published findings from African cohorts in an Asian cohort, the new TB-DM biomarker results reported here for the first time will need to be validated, including in age-matched cohorts as this was not corrected for in this analysis.

We previously identified the ratio of phenylalanine over histidine (Phe/His) as a promising biomarker for TB classification and diagnosis irrespective of DM-status in a South-African patient cohort^7^. This finding is corroborated independently in the current study in a genetically and geographically completely different cohort (Fig. 3B): the Phe/His ratio demonstrated a superior classification capacity for TB versus HC compared to any individual metabolite (AUC: 0.912 [0.850–0.974]), and similar values were obtained for TB-DM patients vs HC (AUC: 0.908 [0.807–1.000]). Furthermore, multiple linear regression analyses showed a relative increase in kynurenine accompanied with decreased tryptophan in TB patients. The ratio between these amino acids (Kyn/Trp) reflects the activity of IDO, which catalyzes the rate-limiting step in the kynurenine pathway of tryptophan catabolism. The results (Fig. 3C) showed that both TB and TB-DM were associated with an increased Kyn/Trp ratio (AUC: 0.838 [0.755–0.922]; AUC: 0.802 [0.682–0.921] respectively), indicative of increased IDO activity. Finally, various amino acids from the urea cycle were found to be divergently affected during TB, including citrulline and arginine (Cit/Arg) which are essential for nitric oxide (NO) production by NO synthase (NOS). TB but not TB-DM was associated with a decreased Cit/Arg ratio compared to HC (AUC: 0.895 [0.834–0.955]), possibly reflecting diminished NO production through NOS in these patients.

### Anti-TB treatment resulted in normalization of diverging metabolites to healthy levels

**Figure 4:**
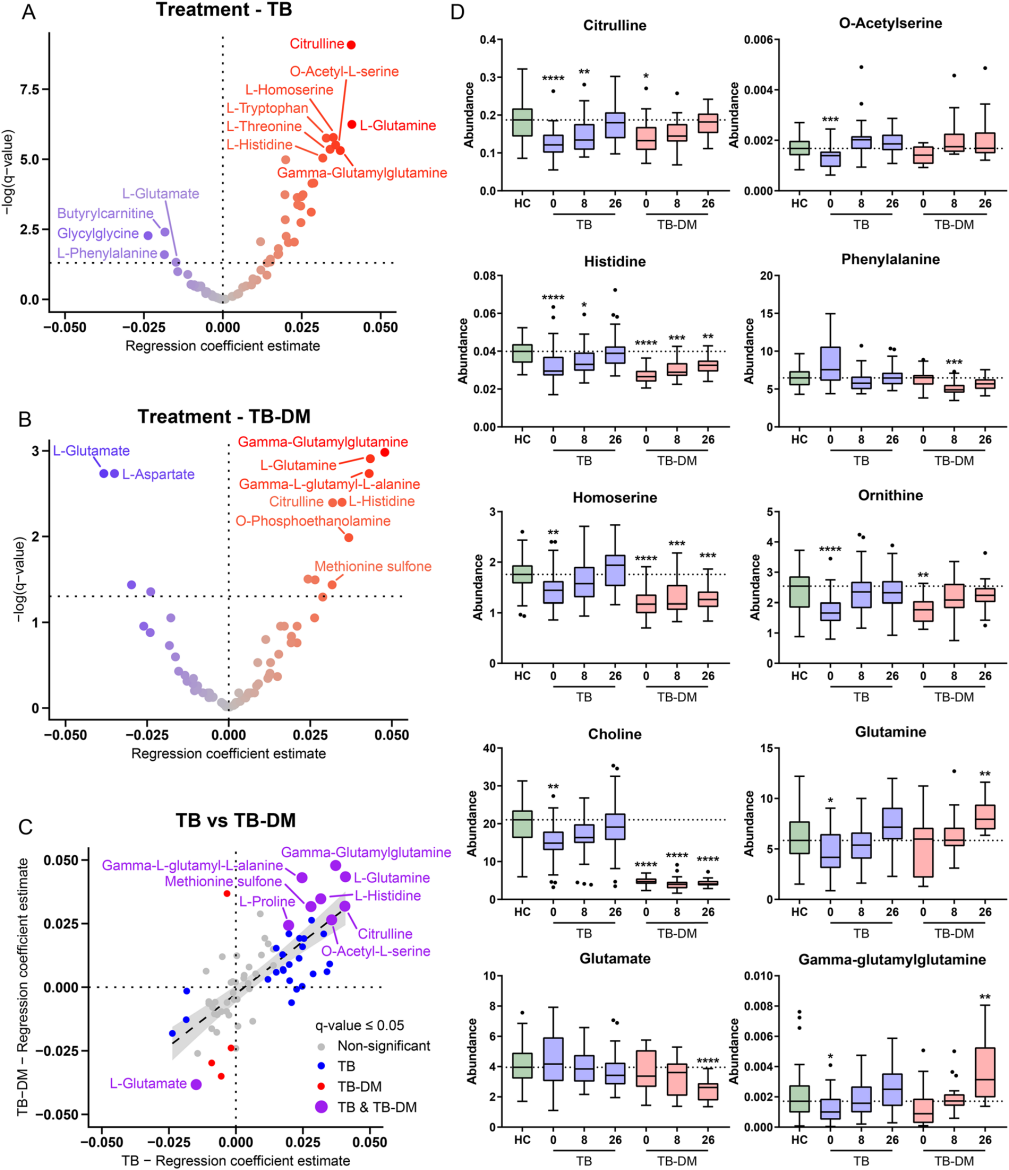
Anti-TB treatment leads to normalization of patient plasma metabolic profiles to HC levels. Linear mixed models were fitted for the effect of antibiotic treatment on metabolite levels in TB patients (**A**) and TB-DM patients (**B**) separately. Resulting -log-transformed *p*-values (*q*-values) are plotted against the regression coefficient estimate for each metabolite. Each dot represents an individual metabolite. Dot color represents direction and size of the regression coefficient. The significance threshold (*q* = 0.05) is displayed as a horizontal dotted line. (**C**) Beta-beta plot of metabolite regression coefficients for the effect of anti-TB treatment in TB patients (x-axis) versus TB-DM patients (y-axis). Each dot represents an individual metabolite. Dot color represents whether the metabolite was significantly affected by anti-TB treatment in TB patients (blue), TB-DM patients (red), both (purple) or not at all (grey). Regression line is displayed as a dashed line with 95% confidence interval. (**D**) Absolute abundance of individual metabolites per group displayed as Tukey’s boxplots. For TB and TB-DM patients metabolite levels are displayed at 0, 8 and 26 weeks post-treatment. Significance differences between HC (n = 48) versus TB (n = 44) or TB-DM (n = 19) patients were determined by Kruskal-Wallis test with post-hoc Dunn’s test. *p = 0.05, **p = 0.01, ***p = 0.001, ****p = 0.0001.

Next, we sought to investigate the effect of anti-TB treatment on the metabolic profiles of TB and TB-DM patients. Plasma samples collected at both ~8 weeks and ~26 weeks after initiation of antibiotic treatment were available and measured for 45/49 TB and 18/19 TB-DM patients, respectively. Successful TB treatment was associated with a significant linear positive effect for 29 metabolites in TB patients (Fig. 4A), while 4 metabolites were downregulated during anti-TB therapy. Many metabolites which were lower in TB patients at diagnosis normalized to HC levels during treatment duration, including citrulline, glutamine, tryptophan, histidine and ornithine, while glycylglycine and phenylalanine were decreased as a result of therapy after previously being upregulated in TB patients’ plasma. Interestingly, 3-methoxytyrosine did not normalize to HC levels during treatment (*q* = 0.928), and could therefore represent a long-lasting TB-associated biomarker.

The metabolic effects of treatment in TB and TB-DM patients showed strong similarities (Fig. 4B,C) as demonstrated by significant positive correlations of metabolite regression coefficients (r^2^: 0.528, *p* = 2.35E^−13^). Metabolites with significant treatment-associated effects in both groups were glutamine, gamma-glutamylglutamine, gamma-glutamylalanine, histidine, citrulline, proline, O-acetylserine and glutamate. Interestingly, the levels of glutamine and gamma-glutamylglutamine were significantly higher in TB-DM patients compared to HC at the end of treatment (*p* < 0.01), while glutamate was simultaneously decreased (*p* < 0.0001) (Fig. 4D). Although treatment resulted in normalization of choline to HC levels in TB patients (*q* = 9.40E^−3^), choline concentrations remained very low in TB-DM patients (*q = *0.756). In addition, levels of glycine, serine, threonine and homoserine did not increase with treatment, further establishing their association with DM in these patients.

As the 2HRZE/4H3R3 treatment regimen is more intensive during the first two months compared to the last four, it is reasonable to expect that a subset of metabolites would react to treatment in a non-linear fashion. Therefore, separate mixed models were fitted for time periods 0 to 8 weeks and 8 to 26 weeks in both disease groups (Fig. S3A,B). Similar to the general linear treatment model, the effects of treatment in TB and TB-DM patients were positively correlated during both 0 to 8 weeks (*r*^2^: 0.739, *p* = 1.04E^−22^) and 8 to 26 weeks (*r*^2^: 0.532, *p* = 1.67E^−13^). When comparing changes in metabolite levels between both time periods, some inverse relationships were observed in both TB and TB-DM patients (Fig. S3C–E). Two metabolites were strongly increased during the first 8 weeks of treatment in both patient populations, namely methionine sulfone and putrescine, while their levels had significantly receded at the end of treatment. In TB patients, 7 metabolites followed an opposite trend with decreased levels at 8 weeks followed by a rise at 26 weeks post-treatment (Fig. S3C), i.e. methionine, glycylproline, asparagine, octenoylcarnitine, lysine, phenylalanine and serine; similar effects were observed in TB-DM patients for all but the latter (Fig. S3D).

### Metabolite associations with TB severity

Finally, we wondered whether some metabolites could be related to TB severity as quantified by CXR score (mild or advanced lesions). To investigate this, CXR was added as a covariate to our initial regression model which was subsequently fitted on TB and TB-DM patients combined. Eleven metabolites showed a significant association with CXR score, however none of these survived FDR-correction, indicating that the statistical power of this dataset was insufficient to accurately assess this question. Nonetheless, to highlight possible trends of metabolic associations with TB severity, we performed classical biomarker analysis using CXR score as identifier and only selected metabolites with univariate t-test statistic *p*-values < 0.01. This resulted in five metabolites with a potential positive association with advanced CXR lesions (Fig. S4), including four acylcarnitines (hexanoylcarnitine, 3-methoxytyrosine, hexadecenoylcarnitine, dodecenoylcarnitine, tetradecenoylcarnitine). These results suggest that while acylcarnitine levels were not strongly associated with TB or TB-DM in the initial regression analysis, some could specifically be affected in individuals with severe disease. These results will have to be validated in studies with more statistical power.

## Discussion

Here, we applied plasma metabolomics to identify differences in amine and acylcarnitine levels associated with TB or TB-DM in a cohort of Indonesian patients at the time of diagnosis as well as during longitudinal follow-up over the course of antibiotic treatment. We identified several potential biomarkers with high AUC values for TB and/or TB-DM diagnosis, which included ratios of citrulline, arginine, phenylalanine and histidine among others. Overall, levels of many amines were decreased in both TB and TB-DM patients at diagnosis compared to HC, while relatively few acylcarnitines were affected. TB patients were further characterized by relatively high levels of several metabolites including the L-DOPA metabolite 3-methoxytyrosine, whereas only putrescine, a polyamine associated with DM^16^, was found to be specifically elevated in TB-DM. This lack of positively correlated metabolites in the TB-DM group was surprising to some extent, as DM is often linked to overnutrition and our previous results have demonstrated that TB-DM patients from a South-African cohort displayed major hallmarks of DM, *e.g*. hyperglycemia, dyslipidemia and elevated branched-chain amino acids^7^. TB-DM patients were further characterized by lower levels of glycine, serine, threonine and homoserine compared to TB patients, which were similarly unaffected by TB treatment. These amino acids are part of the same biosynthetic pathway and have been implicated in the development of non-alcoholic fatty liver disease (NAFLD)^17,18^, a liver disorder commonly associated with DM and insulin resistance^19^.

Importantly, the majority of TB-related metabolites normalized towards HC levels during antibiotic treatment, substantiating their association with active disease. Exceptions to this included choline, which was dramatically lowered in plasma of patients with TB-DM compared to both HC and TB patients and did not change in response to treatment in these patients. We consider it unlikely that this effect is an artifact introduced during measurement or blood collection, as all samples were randomized and blinded before technical analysis and the results were very consistent over multiple independent time points. Decreased choline bioavailability due to reduced intake or gut microbiome dysbiosis have been linked to NAFLD^20,21^ and therefore align with the detected low levels of glycine, serine, threonine and homoserine, all of which similarly did not normalize to HC levels during treatment. However, this result should be interpreted with some caution as similar levels of choline deficiency during either TB or DM have not been reported before to the best of our knowledge.

Our results are in concordance with -and independently validate- earlier metabolic biomarker studies for TB, currently in a cohort from Indonesia. We previously found reduced concentrations of histidine, glutamine, alanine and valine in TB patients from South-Africa combined with high phenylalanine levels^7^, and the described high predictive capacity of the Phe/His ratio as a biomarker for TB regardless of DM-status was confirmed in this current cohort. Similarly, Weiner *et al*. reported lower serum levels of histidine, citrulline, glutamine, gamma-glutamylglutamine, alanine and threonine in active TB patients, while phenylalanine, 3-methoxytyrosine and aspartic acid were elevated^12,22^. Low levels of tryptophan and/or high concentrations of kynurenine have been demonstrated in both TB patients’ sera^22–24^ and pleural fluids^25^. An increased Kyn/Trp ratio is an estimate of enhanced activity of the immunoregulatory enzyme IDO, which was found to benefit *Mtb* infection both *in vitro* and *in vivo*^26^, and showed potential as a biomarker for TB diagnosis in our analysis. This striking agreement between TB metabolomics studies performed using diverse technical platforms as well as patient cohorts from different geographical regions confirms and highlights the robustness of the platforms and resulting data, as well as its potential for diagnosis and prognosis of TB^12^.

TB patients showed decreased levels of citrulline and ornithine, whereas arginine and aspartic acid concentrations were elevated. Furthermore, the Cit/Arg ratio displayed good predictive capacity for TB vs HC, but not for TB-DM vs HC. Citrulline, ornithine, arginine and aspartic acid are important intermediates of the urea cycle, which is responsible for the majority of nitrogen excretion through conversion of toxic ammonia to urea in the liver^27^. At the beginning of the cycle, citrulline is formed from ornithine and ammonia, which subsequently reacts with aspartic acid to form arginine through arginosuccinate. Arginine can then be hydrolyzed by arginase to form urea and ornithine, or be used by NOS leading to the production of NO and citrulline, a balance which has shifted in TB patients as reflected by their relatively decreased Cit/Arg ratio. In mouse models of TB disease, arginase 1 (Arg1) expression in myeloid cells from TB granulomas has been demonstrated to exacerbate disease through substrate competition with NOS^28,29^. Citrulline, however, was shown to fuel antimycobacterial mechanisms of murine macrophages^30,31^ and T-cells^32^ as an alternative source of intracellular arginine, implying that decreased citrulline levels could be detrimental for TB patients. Although the importance of arginase and NO production for the antimycobacterial response in humans remains controversial, *ARG1* was found to be expressed in granulomatous tissue of TB patients^33,34^ and could therefore play a role in TB pathophysiology. Whether the observed changes in arginine, citrulline and ornithine are caused by changes in expression or activity of these enzymes cannot be ascertained from these results, and will have to be addressed in future studies.

Antibiotic treatment led to normalization to HC levels for the majority of TB-associated metabolites in both TB and TB-DM patients, including citrulline, ornithine, histidine and phenylalanine. In contrast, a subset of metabolites specifically changed during the first 2 months of intensive treatment, of which the strongly increased concentrations of methionine sulfone and putrescine were especially striking. Methionine is susceptible to oxidative modification by reactive oxygen species (ROS), and high levels of oxidized methionine are therefore regarded as a marker of oxidative stress^35^. While initial oxidation of methionine leads to the reversible formation of methionine sulfoxide, the second oxidation step to form methionine sulfone is effectively irreversible. Methionine oxidation was found to be associated with drug-induced liver injury^36^, which could be the cause of the observed elevation of methionine sulfone during intensive antibiotic treatment. Surprisingly, although plasma methionine concentrations were appropriately decreased, levels of methionine sulfoxide were not affected by antibiotic treatment in either TB or TB-DM patients. In addition to oxidized methionine, the authors reported elevated levels of gamma-glutamyl dipeptides during various types of liver injury^36^. Gamma-glutamyl dipeptides are formed by gamma-glutamyltransferase (GGT) as byproducts of anti-oxidative glutathione synthesis and therefore reflect oxidative stress. GGT is widely used as a diagnostic marker for hepatic disease and alcohol consumption^37^, and high circulating GGT levels are a risk factor for DM development^38^. Correspondingly, we found that gamma-glutamylalanine and gamma-glutamylglutamine increased with antibiotic treatment, even to levels above HC in TB-DM patients for the latter, which could be indicative of enhanced GGT activity as a result of treatment. Additionally, high levels of putrescine were similarly shown to be associated with hepatotoxicity and antibiotic treatment in animal models^39,40^. Taken together, anti-TB therapy correlated with increased levels of metabolic biomarkers associated with liver injury and oxidative stress, especially during early intensive antibiotic treatment, emphasizing the necessity of liver function monitoring during this period. Since the final blood samples were collected at the end of treatment, it would be informative to measure the abundance of these metabolites sometime after end of therapy in the future to possibly study liver function recovery.

As a result of limitations in patient sampling, several possible confounders of the study need to be discussed. Firstly, TB-DM patients were significantly older compared to both HC and TB patients. Although we attempted to correct for this by including age as a covariate in the regression analyses, it cannot be fully excluded that differences in age explain a proportion of the data variance in TB-DM patients, as levels of threonine, histidine, glycine and serine, for instance, have been demonstrated to decrease with age^41^. Secondly, the average BMI was significantly different between the three groups, which could be correlated with changes in metabolite levels. However, we purposefully chose not to adjust for BMI in our analysis as it is intrinsically associated with the pathophysiology of both TB and DM and consequently its possible effect on patients’ metabolic profiles. Similarly, we could not control for differences in factors such as nutrition and microbiome composition which could also have caused certain specific metabolite alterations. Thirdly, as these measurements were performed on a historic patient cohort no additional control groups could be included. In order to confirm the specificity of the reported metabolic changes for disease, future studies should include DM patients without TB and compare TB to other respiratory or infectious diseases. A recent paper which compared circulating amine and acylcarnitines levels of lung cancer patients to healthy controls reported increased plasma arginine levels while citrulline and glycine were decreased, similar to what we observed for TB, indicating that these could potentially be a reflection of general lung pathology^42^. In contrast, no consistent changes were observed for the other TB-associated amines such as histidine and phenylalanine that we identified. Additionally, a metabolomics study on chronic obstructive pulmonary disease (COPD) showed little overlap with our observations^43^, supporting the specificity of these results for TB. Finally, the use of anti-diabetic medication could have influenced the concentrations of metabolites in the TB-DM group.

In conclusion, TB and TB-DM are associated with marked changes in plasma levels of amine metabolites, which normalize during anti-TB therapy. The presence of TB-DM-specific changes indicates that this comorbidity needs to be considered for the development of diagnostic tests for TB based on levels of metabolic intermediates. This study supports the use of relevant metabolite ratios as potential biomarkers for TB, and it would be of great interest to investigate their possible relation with TB disease progression, severity and treatment outcome in future studies.

## Materials and Methods

### Study subjects

Patients plasma samples included in this study were randomly selected, based on sample availability, from a previously described cohort from Indonesia^44^. In brief, newly diagnosed active pulmonary TB patients were recruited from January 2002 to December 2004 at an outpatient TB treatment center in Jakarta. TB diagnosis was established according to World Health Organization (WHO) criteria, on the basis of clinical presentation and a chest X-ray radiograph (CXR) and confirmed by microscopic detection of acid-fast bacilli in Ziehl-Nielsen-stained sputum smears and positive culture of *Mtb*. Human immunodeficiency virus (HIV)-seropositive patients, patients with cardiac diseases and patients with incomplete data records were excluded. TB patients were classified as having mild-to-moderate TB or advanced TB on the basis of the extent of lesions on CXR. CXR results were divided into lower, middle and upper lung regions, left and right, and abnormalities were scored as ‘mild’ (1 of 6 areas involved), ‘moderate (2 or 3 out of 6 areas) or advanced (more than 3 areas involved)^45^. Diabetes was diagnosed if fasting blood glucose (FBG) was >126 mg/dl, in accordance with WHO criteria at time of recruitment, or by self-reported diabetes. In the same period, healthy individuals matched for sex and age (±10%) and living within the same rukun tetangga (consisting of 15–30 households) were included as control subjects. Controls with diabetes, signs, symptoms, and CXR results suggestive of active TB, a history of anti-TB treatment or incomplete data entry were excluded. HIV status was not tested in the control group, however Indonesia was classified as a country with a low HIV prevalence of ≤0.1% at time of study subject recruitment. Free anti-TB drug treatment was provided to all patients, which consisted of a standard regimen of isoniazid, rifampin, pyrazinamide, and ethambutol (2HRZE/4H3R3) according to the Indonesian national TB program guideline. A subgroup of patients was followed longitudinally, from which blood samples were collected at two and six months after start of treatment. This study was approved by the Ethical Committee of the Faculty of Medicine, University of Indonesia, Jakarta, and by the Eijkman Institute Research Ethics Committee, Jakarta, and written informed consent was voluntarily signed by all patients and control subjects. All research was performed in accordance with relevant guidelines and regulations at time of recruitment.

### LC-MS/MS

Metabolite levels in plasma were measured in individual replicates using two targeted LC-MS/MS platforms. Subject numbers were randomized and run in 5 batches which included a calibration line, QC samples and blanks. QC samples were analyzed every 10 samples, they are used to assess data quality and to correct for instrument response. Blanks are used to check for blank effects.

The amine platform covers amino acids and biogenic amines employing an Accq-Tag derivatization strategy adapted from the protocol supplied by Waters^46^. 5.0 µL of each sample was spiked with an internal standard solution. Then proteins were precipitated by the addition of MeOH. The supernatant was taken to dryness in a speedvac. The residue was reconstituted in borate buffer (pH 8.5) with AQC reagent. 1.0 μL of the reaction mixture was injected into the UPLC-MS/MS system. Chromatographic separation was achieved by an Agilent 1290 Infinity II LC System on an Accq-Tag Ultra column (Waters). The UPLC was coupled to electrospray ionization on a triple quadrupole mass spectrometer (AB SCIEX Qtrap 6500). Analytes were detected in the positive ion mode and monitored in Multiple Reaction Monitoring (MRM) using nominal mass resolution. Acquired data were evaluated using MultiQuant Software for Quantitative Analysis (AB SCIEX, Version 3.0.2).

The acylcarnitine platform covers acylcarnitines as well as Trimethylamine-N-oxide, Choline, Betaine, Deoxycarnitine and Carnitine. 10 µL of each sample was spiked with an internal standard solution. Then proteins were precipitated by the addition of MeOH. 1.0 μL of the reaction mixture was injected into the UPLC-MS/MS system. Chromatographic separation was achieved by UPLC (Agilent 1290, San Jose, CA, USA) on an Accq-Tag Ultra column (Waters). The UPLC was coupled to electrospray ionization on a triple quadrupole mass spectrometer (Agilent 6460, San Jose, CA, USA). Analytes were detected in the positive ion mode and monitored in Multiple Reaction Monitoring (MRM) using nominal mass resolution. Acquired data were evaluated using Agilent MassHunter Quantitative Analysis software (Agilent, Version B.05.01).

The data are expressed as relative response ratios (target area/ISTD area; unit free) using proper internal standards. For analysis of amino acids their ^13^C^15^N-labeled analogs were used. For other metabolites, the closest-eluting internal standard was employed. All internal standards are listed in Supplementary Table 2. In-house developed algorithms were applied using the pooled QC samples to compensate for shifts in the sensitivity of the mass spectrometer over the batches. After quality control correction, metabolite targets complied with the acceptance criteria of RSDqc < 15%.

### Statistical analysis

For multivariate analysis, metabolite measurements were log transformed, mean centered and scaled to standard deviation units. After preprocessing, the data variance associated with disease or sex were investigated by principal component analysis (PCA) and hierarchical clustering. Differences between disease groups were further visualized by fitting three component partial least squares discriminant analysis (PLS-DA) models for each disease group comparison. PLS-DA model evaluation criteria (Q2, R2X, R2Y) were determined after leave-one-out cross validation.

To identify significant differences in metabolite levels between the three groups at diagnosis while correcting for age and sex, the following multiple linear regression model was fitted for each metabolite in separate two-level disease group comparisons (TB vs HC, TB-DM vs HC and TB-DM vs TB):

$$Metabolite={\beta }_{0}+{\beta }_{1}Disease+{\beta }_{2}Age+{\beta }_{3}Sex+\varepsilon $$

where *Disease* = disease group (HC, TB or TB-DM), *Age* = age (years) and *Sex* = sex (male/female).

CXR score was consequently added to the model as a covariate to investigate possible metabolite associations with TB severity.

To analyze and compare the effect of anti-TB treatment on metabolite levels in TB and TB-DM patients, the following linear mixed effect model with random intercept for each individual study participant (*u*_0_*Subject*) was fitted for the TB and TB-DM groups separately:

$$Metabolite=({\beta }_{0}+{u}_{0}Subject)+{\beta }_{1}Treatment+{\beta }_{2}Age+{\beta }_{3}Sex+\varepsilon $$

where *Treatment* = duration of treatment (weeks), *Age* = age (years) and *Sex* = sex (male/female).

Resulting *p*-values were corrected by False Discovery Rate (FDR) using the Benjamini–Hochberg procedure to obtain *q*-values, which were subsequently -log transformed and plotted versus the regression coefficient estimate (*β*_1_) to generate metabolite volcano plots. Alternatively, regression coefficient estimates of two comparisons were plotted against each other (beta-beta plots).

For univariate biomarker analysis, metabolite receiver operating characteristic (ROC) plots and area under the curves (AUCs) were generated for each disease group comparison based on the optimal cut-off as calculated by Youden’s J statistic^47^, defined as the value for which the distance to the diagonal line is maximal. AUC 95% confidence intervals (CI) were computed using 2000 stratified bootstrapping samples. Furthermore, for each group comparison the three metabolites with the highest univariate AUCs were combined in a three parameter metabolic signature. Multivariate ROC curves and AUCs were calculated using a linear SVM algorithm included in the MetaboAnalyst R package (version 1.01.)^48^ after hundredfold repeated random sub-sampling cross validation, during which 2/3 of the samples were used for model training and the remaining 1/3 for model testing.

Statistical analysis of clinical characteristics was performed in SPSS 23 (IBM) by one-way ANOVA (reported *p*-values are the outcome of the F-test) or chi-squared test. Analysis of absolute metabolite concentrations was done in Graphpad Prism 7 by Kruskal-Wallis test with post-hoc Dunn’s test. PCA, PLS-DA, hierarchical clustering, multiple linear regression and linear mixed modeling were performed using R version 3.5.0. and the following packages: mixOmics version 6.3.2^49^, lme4 version 1.1.17^50^, lmerTest version 3.0.1^51^ and ggplot2 version 3.1.0^52^

## Supplementary information

Supplementary Figures and Tables

Suppplementary Table 1

## Data Availability

All data underlying this study are included within the manuscript and its Supporting Information Files.
