# Supplementary material for: Plasma metabolomics in tuberculosis patients with and without concurrent type 2 diabetes at diagnosis and during antibiotic treatment
Source: Sci Rep. 2019 Dec 10;9:18669. doi: 10.1038/s41598-019-54983-5 (PMC6904442; doi:10.1038/s41598-019-54983-5)
Supplement: Supplementary file 1 — Supplementary Figures and Tables [file 41598_2019_54983_MOESM1_ESM.pdf]

Plasma metabolomics in tuberculosis patients with and without concurrent type 2 diabetes at diagnosis and during antibiotic treatment.

Frank Vrieling, Bachti Alisjahbana, Edhyana Sahiratmadja, Reinout van Crevel, Amy C. Harms, Thomas Hankemeier, Tom H. M. Ottenhoff and Simone A. Joosten

Supplementary information

Corresponding author: Dr. Simone A. Joosten

Leiden University Medical Center

Department of Infectious Diseases

Albinusdreef 2

2333 ZA Leiden

The Netherlands

Tel: +31 71 526 4024

E-mail: S.A.Joosten@LUMC.nl

Figure S1

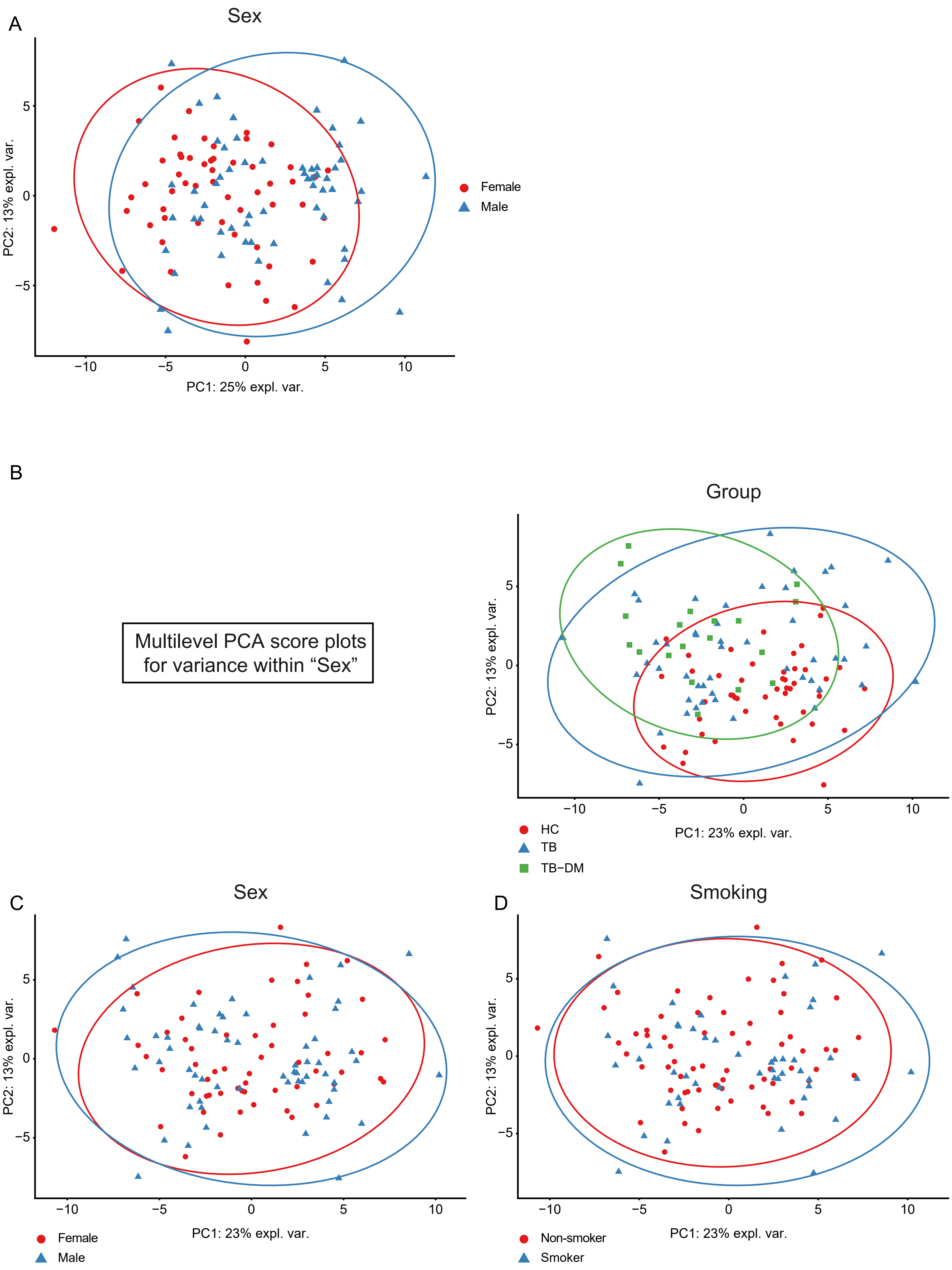
A
Sex
PC2: 13% expl. var.
PC1: 25% expl. var.
Female
Male
B
Multilevel PCA score plots
for variance within “Sex”
Group
PC2: 13% expl. var.
PC1: 23% expl. var.
HC
TB
TB−DM
C
Sex
PC2: 13% expl. var.
PC1: 23% expl. var.
Female
Male
D
Smoking
PC2: 13% expl. var.
PC1: 23% expl. var.
Non-smoker
Smoker

Figure S2

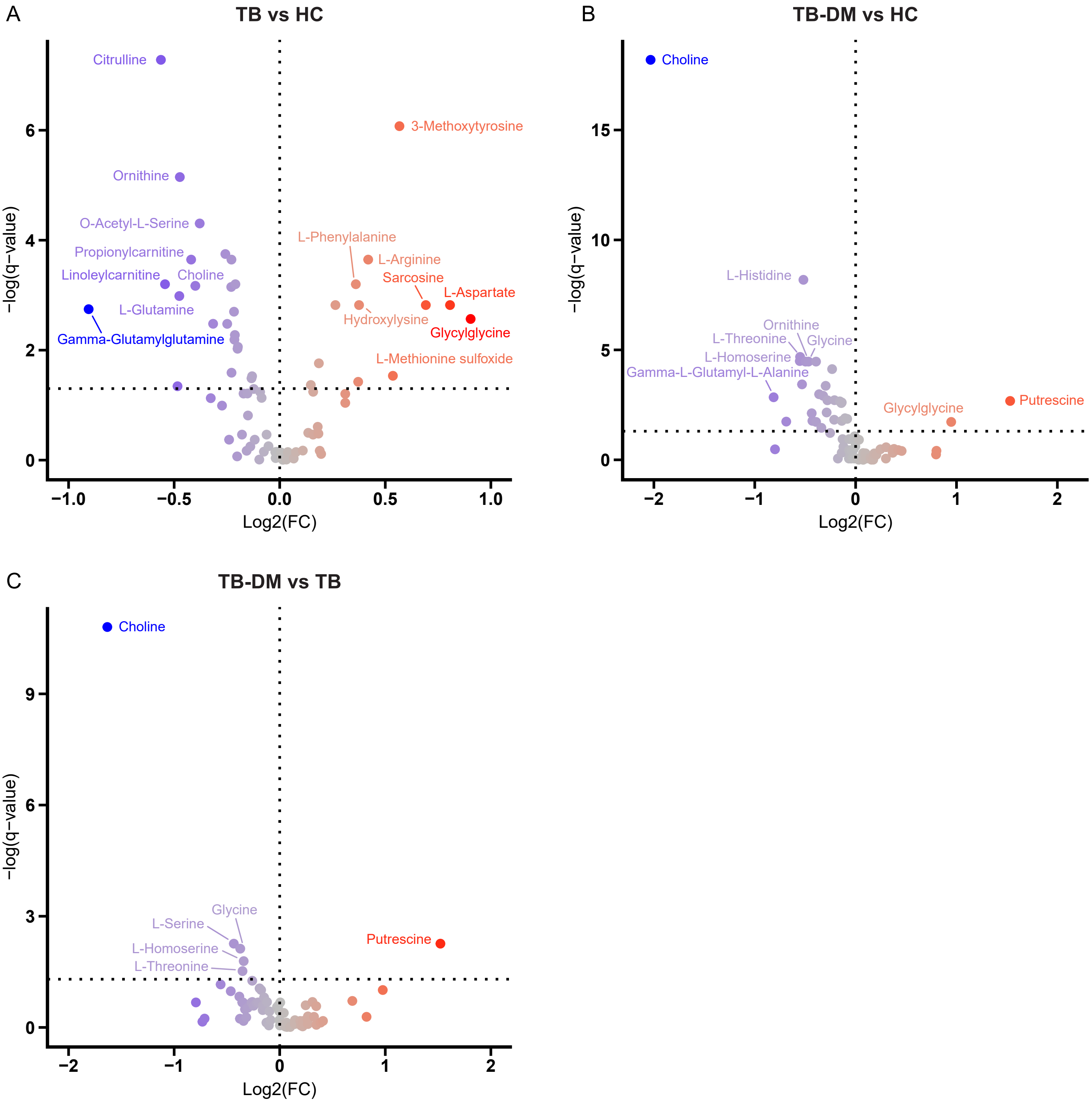
A
TB vs HC
Citrulline
3-Methoxytyrosine
Ornithine
O-Acetyl-L-Serine
L-Phenylalanine
Propionylcarnitine
L-Arginine
Linoleylcarnitine
Choline
Sarcosine
L-Aspartate
L-Glutamine
Hydroxylysine
Glycylglycine
Gamma-Glutamylglutamine
L-Methionine sulfoxide
−log(q−value)
Log2(FC)
B
TB-DM vs HC
Choline
L-Histidine
Ornithine
L-Threonine
Glycine
L-Homoserine
Gamma-L-Glutamyl-L-Alanine
Glycylglycine
Putrescine
−log(q−value)
Log2(FC)
C
TB-DM vs TB
Choline
Glycine
L-Serine
Putrescine
L-Homoserine
L-Threonine
−log(q−value)
Log2(FC)

Figure S3

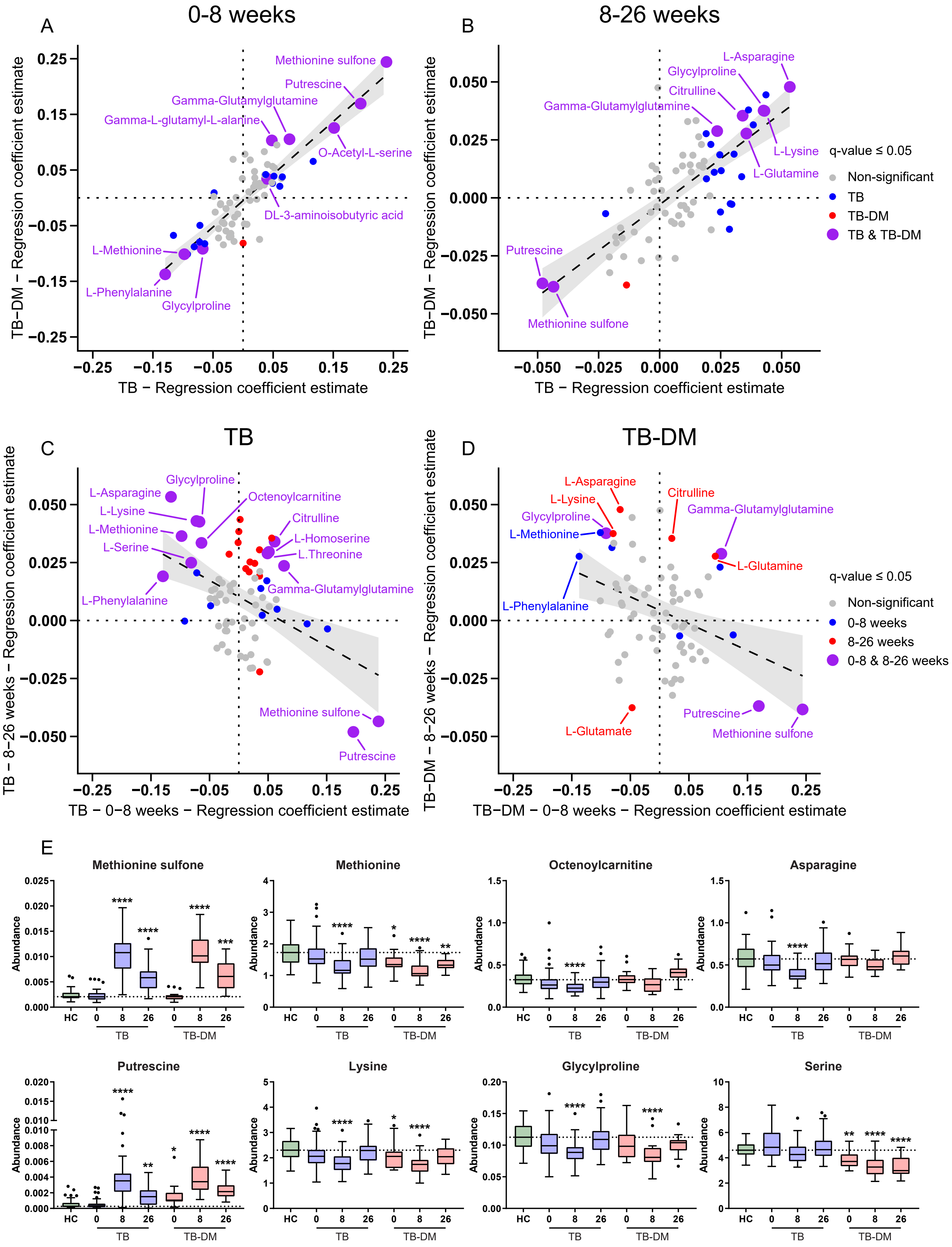
A
0-8 weeks
Methionine sulfone
Putrescine
Gamma-Glutamylglutamine
Gamma-L-glutamyl-L-alanine
O-Acetyl-L-serine
DL-3-aminoisobutyric acid
L-Methionine
L-Phenylalanine
Glycylproline
TB−DM − Regression coefficient estimate
TB − Regression coefficient estimate
B
8-26 weeks
L-Asparagine
Glycylproline
Citrulline
Gamma-Glutamylglutamine
L-Lysine
L-Glutamine
Putrescine
Methionine sulfone
TB−DM − Regression coefficient estimate
TB − Regression coefficient estimate
q-value ≤ 0.05
Non-significant
TB
TB-DM
TB & TB-DM
C
TB
Glycylproline
L-Asparagine
Octenoylcarnitine
L-Lysine
Citrulline
L-Methionine
L-Homoserine
L.Threonine
L-Serine
Gamma-Glutamylglutamine
L-Phenylalanine
Methionine sulfone
Putrescine
TB − 8−26 weeks − Regression coefficient estimate
TB − 0−8 weeks − Regression coefficient estimate
D
TB-DM
L-Asparagine
L-Lysine
Citrulline
Glycylproline
Gamma-Glutamylglutamine
L-Methionine
L-Glutamine
L-Phenylalanine
Putrescine
L-Glutamate
Methionine sulfone
TB−DM − 8−26 weeks − Regression coefficient estimate
TB−DM − 0−8 weeks − Regression coefficient estimate
q-value ≤ 0.05
Non-significant
0-8 weeks
8-26 weeks
0-8 & 8-26 weeks
E
Methionine sulfone
Methionine
Octenoylcarnitine
Asparagine
Putrescine
Lysine
Glycylproline
Serine
Abundance
HC 0 8 26 0 8 26
TB
TB-DM

Figure S4

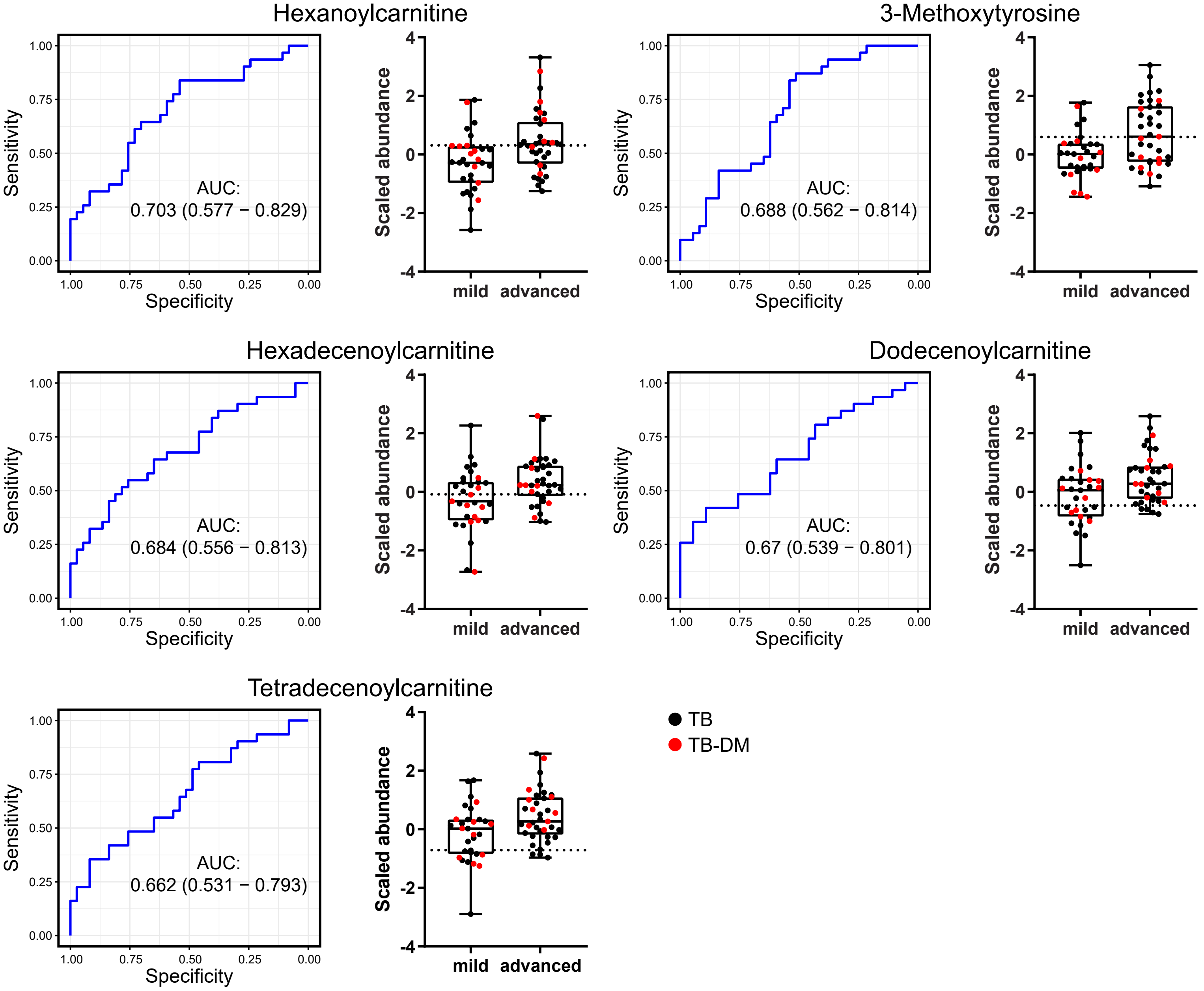
Hexanoylcarnitine
Sensitivity
Specificity
AUC:
0.703 (0.577 − 0.829)
Scaled abundance
mild
advanced
3-Methoxytyrosine
Sensitivity
Specificity
AUC:
0.688 (0.562 − 0.814)
Scaled abundance
mild
advanced
Hexadecenoylcarnitine
Sensitivity
Specificity
AUC:
0.684 (0.556 − 0.813)
Scaled abundance
mild
advanced
Dodecenoylcarnitine
Sensitivity
Specificity
AUC:
0.67 (0.539 − 0.801)
Scaled abundance
mild
advanced
Tetradecenoylcarnitine
Sensitivity
Specificity
AUC:
0.662 (0.531 − 0.793)
Scaled abundance
mild
advanced
TB
TB-DM

Supplementary Table 2:

**Internal standards: amines**
Asn_C13N15
Asp_C13N15
L-ornithine-3,3,4,4,5,5,-d6
L-NT-methyl-d3-L-histidine
Glu_C13N15
Lys C13N15
Ser_C13N15
Beta-alanine-2,2,3,3,-d4
Tyr_C13N15
Gln_C13N15
Thr_C13N15
L-Methionine_C13N15
Arg_C13N15
Ala_C13N15
Val_C13N15
Gly_C13N15
Phe_C13N15
Trp_C13N15
Histamine-α,α,β,β-d4 2HCl
L-2-aminobutyric acid-d6 acid
2-(4-hydroxy-3-methoxyphenyl) ethyl-1,1,2,2-d4-amine
L-Ile_C13N15
Leu_C13N15
**Internal standards: acylcarnitines**
Carnitine-d3 HCl
Betaine-d3 HCl
Deoxycarnitine-d9 HCl
Acetyl-L-carnitine-d3 HCl
Butyryl-L-carnitine-d3 HCl
Octanoyl-L-carnitine-d3 HCl
Octadecanoyl-L-carnitine-d3 HCl

Figure S1: Multilevel PCA for metabolomics data variance within subjects with the same sex improves disease group separation. (A) Score plot of the first two principal components of a PCA model built on the entire dataset and colored for sex with confidence ellipses. Females are displayed as red dots and males as blue triangles. (B-E) Score plots of the first two principal components of a multilevel PCA model for data variance within "Sex", colored for disease group membership (B), sex (C) or smoking status (D).

Figure S2: Volcano plots of linear regression models versus metabolite fold changes. Multiple linear regression models were fitted for each between disease group comparison, and resulting -log-transformed *p*-values (*q*-values) are plotted against $\log_2$-transformed fold changes for each metabolite: TB vs. HC (A), TB-DM vs. HC (B), TB-DM vs. TB (C). Each dot represents an individual metabolite. Dot color represents direction and size of the fold change. The significance threshold (q = 0.05) is displayed as a horizontal dotted line.

Figure S3: Differences between the effect of early (0-8 weeks) versus late (8-26 weeks) anti-TB treatment on patient plasma metabolic profiles. Linear mixed models were fitted for the effect of 0-8 weeks or 8-26 weeks of anti-TB treatment on metabolite levels in TB patients and TB-DM patients separately. (A-B) Beta-beta plots of metabolite regression coefficients for the effect of anti-TB treatment in TB patients (x-axis) versus TB-DM patients (y-axis) for 0-8 weeks (A) and 8-26 weeks (B) of treatment. Each dot represents an individual metabolite. Dot color indicates whether the metabolite was significantly affected by anti-TB treatment in TB patients (blue), TB-DM patients (red), both (purple) or not at all (grey). Regression line with is displayed as a dashed line with 95% confidence interval. (C-D) Beta-beta plots of metabolite regression coefficients for the effect of anti-TB treatment during 0-8 weeks (x-axis) versus 8-26 weeks of anti-TB treatment (y-axis) in TB patients (C) and TB-DM patients (D). Each dot represent an individual metabolite. Dot color represents whether the metabolite was significantly affected by anti-TB treatment during weeks 0-8 (blue), 8-26 (red), both (purple) or not at all (grey). (E) Absolute abundance of individual metabolites per group displayed as Tukey's boxplots. For TB and TB-DM patients metabolite levels are displayed at 0, 8 and 26 weeks post-treatment. Significant differences between HC (n = 48) versus TB (n = 44) or TB-DM (n = 19) patients were determined by Kruskal-Wallis test with post-hoc Dunn's test. * $p = 0.05$, *** $p = 0.001$, **** $p = 0.0001$

Figure S4: Metabolite association with CXR score. ROC curves and AUCs of CXR score classification (mild/advanced lesions) for hexanoylcarnitine, 3-methoxytyrosine, hexadecenoylcarnitine, dodecenoylcarnitine and tertradecenoylcarnitine. Log-transformed and standard deviation unit scaled metabolite abundances are displayed in boxplots. Each dot represents an individual patient (black = TB, red = TB-DM) and the optimal cut-off as determined by Youden's statistic is displayed as a horizontal dotted line.
